# 1192. Containment of Sustained Transmission of Drug-resistant *Acinetobacter baumannii* by Pulsed-Xenon Ultraviolet Disinfection of the Patient Room in the Intensive Care Unit

**DOI:** 10.1093/ofid/ofac492.1027

**Published:** 2022-12-15

**Authors:** Keita Morikane, Jun Yakuwa, Masaki Nakane

**Affiliations:** Yamagata University Hospital, Yamagata, Yamagata, Japan; Yamagata University Hospital, Yamagata, Yamagata, Japan; Yamagata University Hospital, Yamagata, Yamagata, Japan

## Abstract

**Background:**

No-touch environmental disinfection has been highlighted in the last decade to control transmission of multidrug-resistant Gram-positive organisms including MRSA and VRE, but its effectiveness to control gram-negative bacteria has not been well examined. Also, its effectiveness outside the US healthcare setting is seldom reported.

**Methods:**

This study was conducted in the intensive care unit (ICU) of Yamagata University Hospital, a 637-bed tertiary referral hospital. Sporadic acquisition of two drug-resistant *Acinetobacter baumannii* (2DRA) began in late 2013. Despite various infection control practices including hand hygiene promotion and intensified manual terminal cleaning, transmission of this pathogen continued. In February 2018, pulsed-xenon ultraviolet (PX-UV) disinfection was added. The study periods were defined as follows: the baseline period (August 2016 to January 2018, intensified manual cleaning) and the intervention period (February 2018 to December 2021, addition of PX-UV). Throughout the study periods, all patients were regularly screened for 2DRA to detect acquisition of those pathogens in the ICU.

**Results:**

The incidence of newly acquired 2DRA significantly declined over time (4.45 per 1,000 patient days in the baseline period to 1.20 in the intervention period, relative risk (RR): 0.27, 95% confidence interval: 0.12-0.61). Notably, horizontal transmission of 2DRA was completely contained, not only in the ICU but also throughout the hospital (Figure).

Newly acquired 2DRA events (all wards, monthly)

**Figure:**
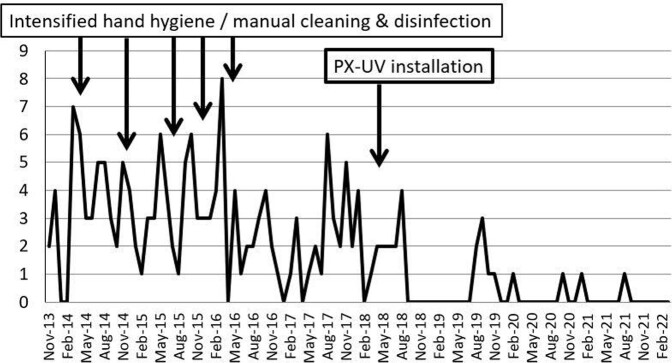

**Conclusion:**

Adding PX-UV after through manual terminal cleaning is effective in controlling acquisition of 2DRA in the ICU patients and led to termination of transmission of 2DRA in our hospital. The effectiveness of PX-UV in controlling gram-negative MDROs in the non-US healthcare settings is suggested.

**Disclosures:**

**All Authors**: No reported disclosures.

